# Is it possible to predict neurosensory alterations in impacted lower third molar removal based on preoperative imaging procedures? A prospective cohort study

**DOI:** 10.4317/medoral.26056

**Published:** 2023-11-22

**Authors:** Fabian Pérez-González, Luis Sánchez-Labrador, Jorge Cortés-Bretón-Brinkmann, Luis Miguel Sáez-Alcaide, Santiago Bazal-Bonelli, Cristina Madrigal-Martínez-Pereda, Juan López-Quiles

**Affiliations:** 1 Department of Dental Clinical Specialties, Faculty of Dentistry, Complutense University of Madrid, Spain; 2 Surgical and Implant Therapies in the Oral Cavity Research Group, Complutense University of Madrid, Spain

## Abstract

**Background:**

Surgical extraction of the lower third molar (LTM) may trigger neurosensory injury of the inferior alveolar nerve, making extraction a real challenge. This study set out to assess whether is it possible to predict neurosensory alterations from preoperative imaging.

**Material and Methods:**

A total of 99 patients underwent 124 impacted lower third molar (ILTM) surgeries. Prior to surgery, panoramic and CBCT images were evaluated in an attempt to predict a neurosensory disturbance. Preoperative data (ILTM position, panoramic radiograph signs, inferior alveolar nerve (IAN) location and its contact with the ILTM roots) and intra/postoperative findings (extraction difficulty and sensitivity alterations) were recorded. Descriptive and bivariate data analysis was performed. Statistical comparison applied the chi-square test, Fisher test, and one-way ANOVA test. Statistical significance was established with a confidence interval (CI) of 95%.

**Results:**

In 4.03% of cases, patients experienced neurosensory alterations. Of 124 ILTM positions in panoramic radiographs, 76 cases were considered to exhibit a potential neurosensory risk as they presented two or more types of superimposed relationships between ILTM and mandibular canal. Of these, alterations were reported in only three cases (3.95%). Of the 48 remaining ILTM images presenting only one sign, neurosensory alterations were observed in two cases (4.17%). No permanent alterations were recorded in any of the five cases observed.

**Conclusions:**

Within the limitations of the present study, prediction of neurosensory alterations prior to ILTM extraction by means of preoperative imaging did not show a significant statistical correlation with post-surgical incidence. Nevertheless, interruption of the canal´s white line (ICWL) or a diversion of the canal (DC) may predict an increased risk of IAN injury.

** Key words:**Impacted lower third molar, inferior alveolar nerve, sensitivity alterations, panoramic radiographs, cone bean computed tomography.

## Introduction

Surgical management of impacted lower third molars (ILTM), whether for prophylactic or symptomatic reasons, is one of the most common procedures performed by oral and maxillofacial surgeons (1). According to the literature, the prevalence of ILTM ranges between 3% and 77% (2). 

Inferior alveolar nerve (IAN) injury is one of the most critical complications that can occur as a result of ILTM extraction, causing neurosensory impairment of the lower lip and chin area. This will impact on the patient’s quality of life.

The complication affects 0.4-5.5% of patients and is usually temporary but can occasionally cause long-term or even permanent symptoms (3), although permanent impairment is relatively infrequent (less than 1%) (1,4).

The literature reports numerous variables associated with higher or lower risks of causing IAN damage during ILTM surgical management. These include the patient’s age, the surgeon’s experience, impaction depth, method of anesthesia, tooth morphology, root proximity to the IAN canal, size of the cortical defect in the inferior alveolar canal (assessed by computed tomography), and operative IAN exposure. So, careful pre-operative evaluation of these factors is of paramount importance when contemplating LTM surgery (4,5).

**Figure 1:**
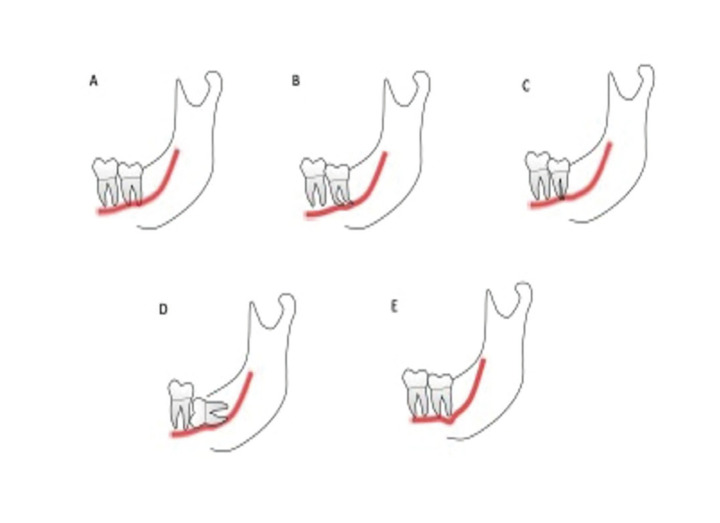
Panoramic superimposed relationships between the ILTM and the mandibular canal related to higher risk of nerve injury during ILTM extraction. A) Darkening of the roots (DR); B) Deflection of the roots (DFR); C) Narrowing of the roots (NR); D) Interruption of the canal´s white line (ICWL); E: Diversion of the canal (DC).

While Rood and Shehab (6) in 1990 described seven types of superimposed relationships between the ILTM and the mandibular canal visible in panoramic radiographs, IAN injury has been found to be significantly related to only five of them: radiographic darkening of the roots (DR), deflection of the roots (DFR), narrowing of the roots (NR), interruption of the canal´s white line (ICWL), and diversion of the canal (DC) (Fig. 1) (4,5).

The presence of two or more superimposed relationships indicates a close relationship between ILTM and IAN, pointing to a higher risk of IAN exposure or injury (4).

According to the European Academy of DentoMaxilloFacial Radiology, CBCT imaging should only be used when surgeons need to address a very specific clinical enquiry in an individual case that cannot be answered by conventional (panoramic and/or intraoral) imaging (7). Furthermore, when panoramic images raise doubts about the relationship between third molar roots and the mandibular canal, Computed Tomography (CT) or Cone Beam Computed Tomography (CBCT) may be used to verify the relationship in three dimensions (3D) (8-10).

The aim of this prospective study was to evaluate whether is it possible to predict neurosensory alterations based on preoperative imaging procedures (orthopantomography and CBCT) prior to surgical ILTM removal.

- Abbreviations

LTM: Lower Third Molar; ILTM: Impacted Lower Third Molar; IAN: Inferior Alveolar Nerve; CI: Confidence Interval; ICWL: Interruption Of the Canal’s White Line. DC: Diversion Of the Canal; DR: Darkening Of the Roots; DFR: Deflection Of the Roots; NR:Narrowing Of the Roots; CtT: Computed Tomography; CBCT: Cone Beam Computed Tomography; 3D: Three Dimensions; STROBE: Strengthening The Reporting of Observational Studies in Epidemiology.

## Material and Methods

- Study design and sample

This prospective clinical cohort study included a total of 124 ILTM extractions in 99 patients performed at the Postgraduate Oral Surgery Clinic at the Faculty of Dentistry, Complutense University of Madrid, Spain, between September 2019 and September 2021. All patients were provided with full information about the purpose of the study and the procedures involved and gave their informed consent to take part. The study was conducted following STROBE (Strengthening the Reporting of Observational Studies in Epidemiology) guidelines (11). All procedures involving the human participants fulfilled ethical standards established by institutional and/or national research committees in accordance with the 1964 Helsinki declaration and subsequent amendments. The study protocol was assessed and approved by the Research Ethics Committee at the San Carlos Hospital of Madrid, Spain in August 2019 (Registration Code Nº 19/331-E).

Inclusion criteria were: patients aged over 18 years; no relevant systemic diseases (American

Society of Anesthesiologists classification ASA I and ASA II); presenting symptoms or indications for ILTM extraction; patients with at least one panoramic radiographic sign of a close relation between ILTM and IAN, therefore requiring a CBCT. Exclusion criteria were: disease or medication prohibiting oral surgery, pregnancy or lactation, patients unable to attend follow-up visits, and ILTM without panoramic radiographic signs of a close relation with the mandibular canal.

The surgical procedure was performed by two oral surgeons (F.P-G and L.S.-L). Local anesthetic consisted of 4% Articaine and 1:100,000 adrenaline for the inferior alveolar, lingual, and buccal nerves (Ultracaine,® Normon SL, Madrid, Spain). An intrasulcular incision was made on the ramus from the lower first molar with a vertical releasing incision; a mucoperiosteal flap was elevated. A round tungsten No. 8 carbide bur with a surgical handpiece was used to perform bone removal and, if necessary, to section the third molar. After ILTM extraction, the bony edges were smoothed, and the socket washed with saline solution. Finally, the flap was sutured with simple interrupted sutures using 5.0 Supramid (Proclinic®, Zaragoza, Spain). All patients were prescribed 1g of amoxicillin one hour before the surgical procedure (12) and anti-inflammatory 400 mg Ibuprofen every 6h for 4 days (13) or in combination with 500 mg acetaminophen (8h for 5 days) for pain relief (14). The patients were recalled on day seven for suture removal and neurosensory analysis (15). In case of neurosensory disturbance reported at follow-up visits, patients were treated with a combined multivitamin B-complex (B1, B6, and B12. Hidroxil©, Almirall SA, Barcelona, Spain) and revised every 15 days (16).

- Data collection

A case history for each participant was created, including clinical history (age, sex, preoperative variables describing ILTM position, and radiographic observations), which surgeon performed the extraction, as well as intra- and post-operative variables.

**Figure 2:**
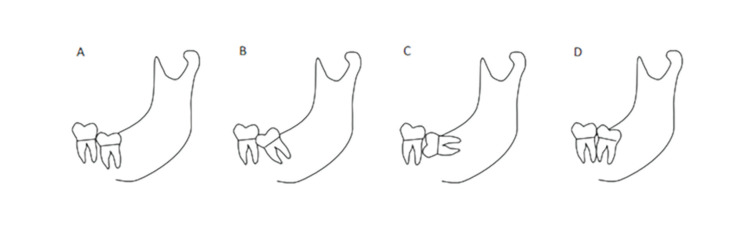
Third molar position according to Winter´s classification. A) Vertical. B) Mesioangular. C) Horizontal. D) Distoangular.

All radiographic images - both panoramic radiographs and CBCT scans - were evaluated prior to the surgical extraction by two experienced oral surgeons (J.C-B.B, J.L-Q), recording the following: location and position of each ILTM according to Winter´s classification (17) (Fig. 2); risk of IAN injury due to the presence of a single sign of superimposed relationship between ILTM and mandibular canal (moderate risk) visible in panoramic radiographs; risk of IAN injury due to the presence of two or more signs (high risk). Panoramic radiographic signs were classified as DR, DFR, ICWL, DC or NR.

At the same time, an exhaustive study of the CBCT was carried out to assess the intimate relationship or otherwise of ILTM roots and IAN. An intimate relationship was defined as the IAN appearing to be in contact (1mm) with the ILTM roots or, failing that, located between the roots.

The Kappa coefficient was used to evaluate the concordance between the two evaluators. Any doubts arising were resolved by discussion and consensus among the research project team members.

Intra- and post-operative variables were also recorded including extraction difficulty and adverse effects during and after ILTM extraction (18).

Finally post-operative type and duration of any IAN injuries were recorded. Neurosensory disturbances, if any occurred, were monitored for twelve months. If the disturbance persisted throughout this period, it was decided that for the purposes of the study, this occurrence would constitute permanent injury.

- Statistical analysis

Statistical analysis was conducted at the Data Processing Center of the Complutense University of Madrid by an independent statistician. Data were analyzed with SPSS* Statistics 28.0 software (SPSS® inc, Chicago IL, USA). This consisted of univariate analysis (mean, standard deviation, median) and bivariate analysis to relate different variables and to analyze data variations using the chi-squared test, Fisher test, and ANOVA test. Statistical significance was established with a confidence interval (CI) of 95%. (*p*<0.05).

## Results

A total of 99 patients (4.73%) were selected from an initial sample of 2,091 patients attending the Oral Surgery Service. All underwent ILTM surgery between September 2019 and September 2021. A total of 124 LTM extractions were assessed. The mean age of patients was 28.45 ± 10.65 years. The sample was made up of 60 women (60.60 %. CI 95%: 52.5%-69.5%) and 39 men (39.39%. CI 95%: 30.5%-47.5%).

All the ILTMs were extracted without any intra-operative complications. Information about patients, position of IAN, ILTM, and their relationship assessed by means of CBCTs are shown in Table 1. The inter-reviewer Kappa statistic between the two independent reviewers was 0.915±0.045 (CI 95%: 0.826 to 1.005), so the intervention of a third reviewer for consensus purposes was not needed.

**Table 1:**
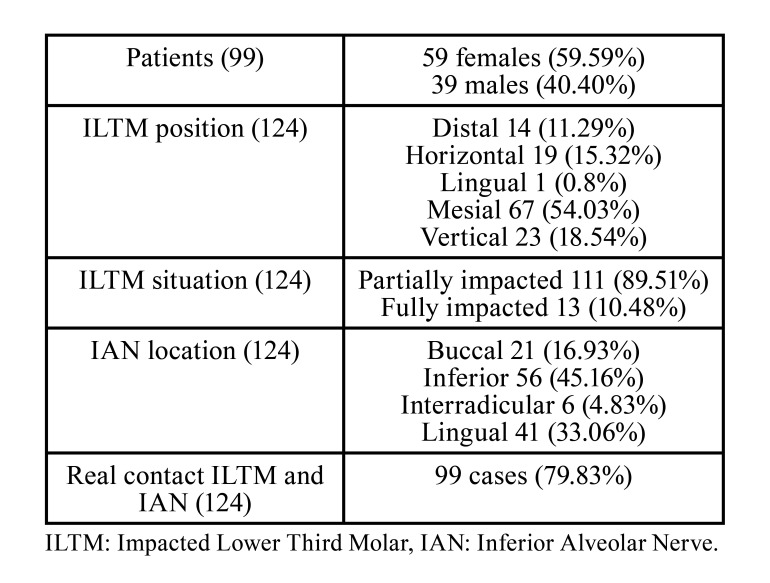
Data information about patients, ILTM and IAN position and its real contact or not on CBCT.

Regarding panoramic radiographic signs, the most frequent were ICWL (74.2%. CI 95%: 66.0%-81.3%) and DR (74.2%. CI 95%: 66.0%-81.3%), followed by DC (17.7%. CI 95%: 11.8%-25.2%) and DFR (4.0 %. CI 95%: 1.6%-8.6%). No NR signs were recorded. According to the number of signs observed in panoramic radiographs, 48 cases presented a single sign (38.7%. CI 95%: 30.5%-47.5%); 64 cases showed two-signs (51.6%. CI 95%: 42.9%-60.3%) and 12 cases presented a combination of three-signs (9.7%. CI 95%: 5.4%-15.8%).

**Figure 3:**
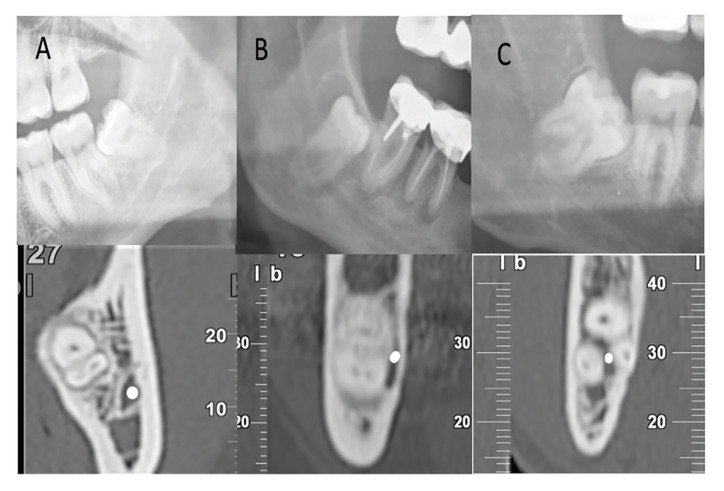
Panoramic image of ILTMs and their corresponding CBCT images. A) Upper image: Panoramic radiograph showing DR, ICWL and DC. Lower Image: CBCT showing no contact between ILTM and IAN (located buccal to the ILTM). B) Upper Image: Panoramic radiography showing DR, ICWL and DC. Lower image: CBCT showing real contact between ILTM and IAN (located lingually to the LTM). C) Upper Image: Panoramic radiography showing DR and ICWL Lower image: CBCT showing an interradicular position of the IAN between ILTM roots.

No significant statistical correlation was found between the presence of single radiographic signs and direct contact observed in the CBCT (*p*=0.062); or the number of radiographic signs and direct contact (*p*=0.686). The main statistical results are summarized in Table 2.

**Table 2:**
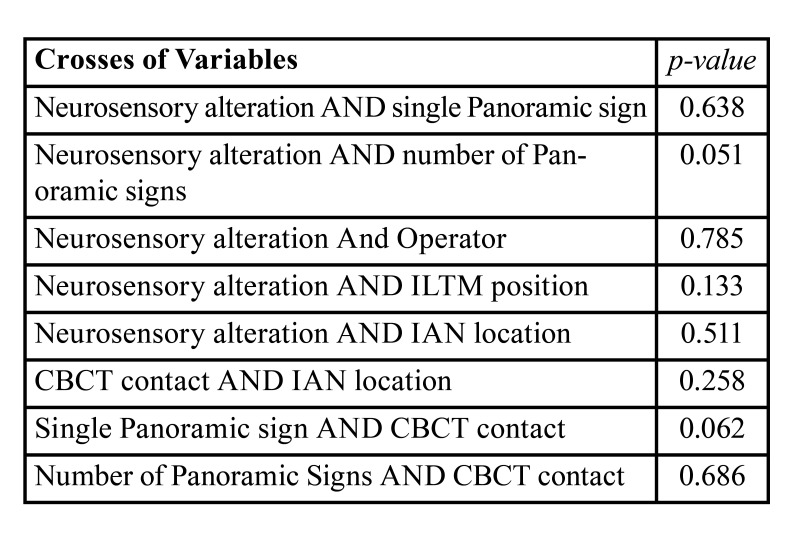
Summarized statistical results.

Furthermore, direct contact between ILTM roots and IAN (verified by means of CBCT imaging) appeared in 99 cases (79.8%. CI 95%: 72.1%-86.2%) (Fig. 3); 62 cases out of 76 (81.57%) that presented a combination of two or three signs in the panoramic radiograph showed direct contact by means CBCT, while the remaining 37 cases out of 48 presented only one sign (77.08%).

Of the 124 ILTM extractions, patients experimented neurosensory alterations in five cases (4.0%. CI 95%: 1.6%-8,6%) (Table 3). Of the 76 cases presenting two or three types of superimposed relationships between the ILTM and the mandibular canal (therefore assessed preoperatively as ‘high risk’) only three cases (3.95%) reported alterations. Of the 48 remaining ILTMs presenting only one sign, neurosensory alterations were observed in two cases (4.17%).

No permanent alterations were recorded in either of the two groups. Four patients (3.23%) reported hypoesthesia, which disappeared during the first two weeks after surgery. In one case (0.81%), the patient described a hyperesthesia sensation that resolved in the third month after surgery.

In light of these results, no statistically significant relationships were identified between either the position of the ILTM according to Winter classification (*p*=0.133) or the position of the ILTM (*p*=0.511) and neurosensory alterations.

No statistically significant relationship was observed between the presence of a single radiographic sign and a higher risk of neurosensory alteration (*p*=0.638), or between the number of signs and neurosensory alteration (*p*=0.051).

However, a tendency towards major risk was observed when the ILTM presented three signs of superimposition and the CBCT confirmed a lingual position. Out of these 12 cases, six presented a lingual nerve position with neurosensory alterations in two cases (33%) (Table 4).

**Table 3:**
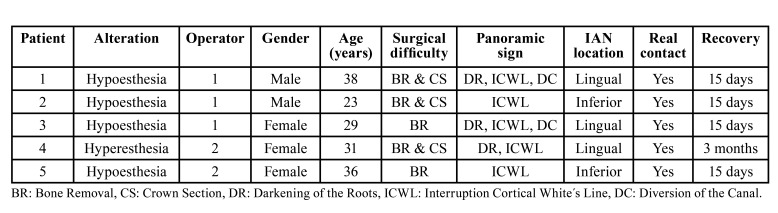
Data of the patients with neurosensory disturbance after ILTM extraction.

**Table 4:**
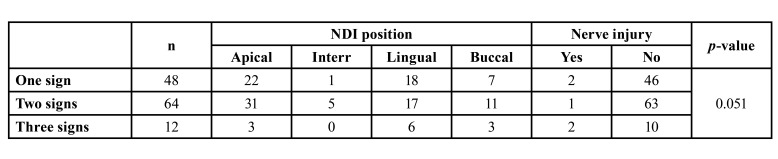
Relationship between the IAN position in the CBCT, number of panoramic radiographic signs and nerve injury.

## Discussion

The aim of this prospective study was to assess the efficacy of preoperative imaging procedures (orthopantomography and CBCT) for predicting neurosensory alterations in ILTM removal surgery.

A recent systematic review found that the use of CBCT analysis was no more effective than panoramic radiography analysis for avoiding neurosensory disturbances (19). Nevertheless, the authors stated that further studies were needed to confirm their findings. So, for the time being, panoramic radiography for anatomical assessment of IAN location and position may be considered the gold standard method of diagnosis and treatment planning for ILTM extraction (20). In CBCTs, the present study identified direct contact between ILTM roots and IAN in 99 cases out of 124.

In light of the present results, the type of radiographic sign and/or the number of signs of superimposed relationships indicating a close relationship between third molar and IAN observed in panoramic radiographs did not show a significant statistical correlation with postoperative neurosensory alteration.

Nevertheless, it would appear that not all the different radiographic signs have the same clinical value. Hasagawa *et al*. have found that ICWL and DC in CBCTs are associated with loss of canal cortication (21). They also argued that these two signs are crucial in predicting an increased risk of IAN injury during LTM removal. In the present study, all patients with neurosensory alterations presented ICWL; percentagewise, a higher incidence was observed among those cases presenting DC (9.09 %; two cases out of 22). These findings also concur with a recent systematic review by Kang *et al*. (22), whereby DC was related to a high percentage of IAN injury (44.4%). At the same time, it should be noted that this radiographic finding is not very common, which is the main reason why it does not exhibit statistical power.

When two signs or three signs appeared in a panoramic radiograph (therefore considered ‘high risk’), the CBCT verified an intimate relationship in 81.57% of cases. This percentage decreased to 77.08% in cases showing a single radiographic sign. In any case, close contact between the LTM and the IAN does not appear to be the only significant factor in predicting nerve injury. In this sense, future studies should also evaluate additional factors that could influence neurosensory alterations such as the surgeon's experience, the surgical technique used, the patient`s age or/and the patient's own anatomical alterations.

In five cases (4.03%), the patient experimented neurosensory disorders after surgery. These results agree with De Toledo *et al*. and Albuquerque *et al*. who recorded values below 10% of patients (19,23).

Contrary to what might be expected, only three cases of neurosensory disorder were recorded out of 76 patients (3.95%) initially classified as ‘high risk,’ while the other two cases corresponded to ILTMs classed as ‘moderate risk’ (out of 48 cases; 4.17%). All five patients underwent completely recovery without further surgical treatment. Four patients affected with hypoesthesia recovered within two weeks, while the remaining patient recovered within three months.

It should be noted that assessment of neurosensory disturbance is based on the patient’s own perception, and so is entirely subjective (24). For this reason, all the data published in reports of neurosensory complications suffer an inherent risk of bias derived from patient subjectivity (25).

Considering the present results, neither ILTM position (following Winter classification) nor IAN position showed any relationship with neurosensory alteration.

Regarding the position of the IAN, in three cases of neurosensory disorder out of the five observed, the IAN was situated in lingual position; in the other two cases the IAN was apical to the ILTM. These observations concurred with Awad *et al*. who recorded the IAN in lingual position in 60% of cases of neurosensory injury (26). Moreover, a higher risk of IAN injury can be expected when the ILTM presents deeper impaction (22). In the present study, three out of the five cases with nerve impairment presented deep impactions requiring extensive bone removal involving crown and root section.

A tendency toward major risk was observed whenever the ILTM presented three signs of superimposition and the CBCT confirmed a lingual position. Out of 12 cases, six presented lingual nerve positions, and 2 of these (33%) coincided with neurosensory alteration. However, it is impossible to draw a firm conclusion given the small number of cases presenting three signs. Nevertheless, when these circumstances arise, the surgeon should consider alternative treatment options such as coronectomy or orthodontic traction.

The coronectomy technique avoids extraction of the entire third molar; dental section separates the crown from the roots, leaving the roots in place. This has been found to reduce the incidence of nerve injury in comparison with complete ILTM removal (27). Nevertheless, to date there is insufficient evidence to draw definite conclusions about which technique - coronectomy or complete surgical extraction - will best avoid neurosensory alterations (28).

Orthodontic third molar traction by means of brackets or mini bone screws also avoids iatrogenic IAN injury, although the technique requires a longer treatment time and there is little literature evaluating the technique (29).

## Conclusions

According to the present study, prediction of neurosensory alterations before surgical extraction of ILTM by means of preoperative imaging procedures did not show a significant statistical correlation with post-operative incidence. Only five cases (4.03%), all of which exhibited ICWL, presented neurosensory disorders after surgery. All underwent complete recovery without further surgical treatment.
